# Co-Design in Electrical Medical Beds with Caregivers

**DOI:** 10.3390/ijerph192316353

**Published:** 2022-12-06

**Authors:** Davide Bacchin, Gabriella Francesca Amalia Pernice, Leonardo Pierobon, Elena Zanella, Marcello Sardena, Marino Malvestio, Luciano Gamberini

**Affiliations:** 1 Department of General Psychology, University of Padova, 35131 Padova, Italy; 2 Human Inspired Technology (HIT) Research Centre, University of Padova, 35121 Padova, Italy; 3 Malvestio Spa, Villanova di Camposampiero, 35010 Padova, Italy

**Keywords:** usability, focus group, medical beds, health technology, co-design, healthcare

## Abstract

Among the plethora of instruments present in healthcare environments, the hospital bed is undoubtedly one of the most important for patients and caregivers. However, their design usually follows a top-down approach without considering end-users opinions and desires. Exploiting Human-centered design (HCD) permits these users to have a substantial role in the final product outcome. This study aims to empower caregivers to express their opinion about the hospital bed using a qualitative approach. For a holistic vision, we conducted six focus groups and six semi-structured interviews with nurses, nursing students, social-health operators and physiotherapists belonging to many healthcare situations. We then used thematic analysis to extract the themes that participants faced during the procedures, providing a comprehensive guide to designing the future electrical medical bed. These work results could also help overcome many issues that caregivers face during their everyday working life. Moreover, we identified the User Experience features that could represent the essential elements to consider.

## 1. Introduction

There are many instruments and devices present in hospitals. Many have become obsolete over time, but one will never go into disuse: the medical bed. The development of the modern hospital bed started between 1815 and 1825, introducing adjustable rails. At the beginning of 1900, the beds began to present three sections to allow both head and feet to be elevated [1]. In 1956, the bed’s equipment started to include electrical functions. Nowadays, almost all medical beds present side rails with control pads, three electrically movable sections, adjustable height and other features. The guiding concept behind this evolution was the creation of a tool to provide comfort to patients and reduce caregivers’ workload.

Most of the time, research in this field has approached new feature development starting from designers’ ideas. Many examples in literature follow this path, testing new features such as integrated toilets [2], systems to prevent bedsores [3], bedside angle measuring devices [4], and bed movers [5], to name just a few. Nowadays, human-centred design (HCD) is replacing this approach. In this new technology development vision, the innovation process actively involves stakeholders, empowering them by exploiting participatory methods from ideation to testing a new product. The human-centred design approach offers many advantages. One of the most important is the possibility of addressing real user problems and permitting people to have a substantial role in the design process outcomes [6]. Human–Computer Interaction (HCI) and Design Thinking (DT) are two slightly different design approaches that share this vision of understanding and observing users.

Both start with discovering users’ problems. The difference between the two is that HCI aims to understand users’ requirements, and DT instead stresses the concept of building empathy with them. The HCI process generally emphasizes the solutions’ analysis, evaluation and testing, while DT focuses on profound observation and inquiries [7]. However, HCI has a more systematic vision of the design process due to its exploitation of golden rules and guidelines (i.e., Nielsen’s 10 Heuristics [8]). The first step in the HCI process is the “what is wanted” phase. In this phase, researchers investigate users to provide information about their needs using tools such as interviews (e.g., Focus Groups), user behaviour recording, document analysis and direct observation. Then the “analysis” step collects and organizes the results to provide valuable insights for the “design” phase and generate solutions. The designers often exploit early prototypes to test the users’ interaction efficacy with the product and enhance its usability. This evaluation phase permits the study of issues with the prototype in the early stage of development and can be performed many times, creating an iterative process. The subsequent correction of the problems and assessing the prototype’s efficacy and functionality bring the product to its implementation and deployment in the market [9].

### 1.1. Human-Centred Design in Healthcare Environments

The literature presents many examples of the exploitation of HCD. Indeed, many fields of activity experience the benefits of this approach, such as industry [7,10], education [11], Internet of Things technologies [12], transport and automotive [13,14], websites [15], applications for people with disabilities [16] and many others. Among these, one field that is receiving colossal attention is healthcare. For example, Harte and colleagues [17] proposed a structured methodology that could help designers follow HCD principles to consider user needs while maintaining a short development time. Their method was composed of three phases: construction of a use case document, expert usability inspection and end-user testing. They applied the method to a fall detection and prevention system, indicating that this methodology could be a good support for designing connected health devices. Moreover, public health projects are applying the HCD approach. In their interesting review, Bazzano and colleagues [18] indicated four global health contexts that exploited participatory design:

Disease management, serious and chronic health conditions (e.g., dementia, cancer), health system and care management (e.g., HIV patients, vaccine safety), infectious disease prevention/care (e.g., hand hygiene, health care workers), primary intervention and health behaviour/education (e.g., contraception, infant mortality) 

In our study, we were interested in applying HCD in specific healthcare locations: hospitals and elderly retirement homes. These environments represent a challenge for HCD studies because of their complexity. Indeed, the literature presents some examples of studies that try to simplify and re-design them [19,20]. The employees that have to manage and face this complexity are mainly caregivers, often involved in HCD processes (e.g., discovering needs, end-user testing) for research technologies. In some cases, they participate in the early phases of a device’s development, giving information about user requirements for specific technologies [21,22,23,24,25], while in other cases, they test the developed devices [25,26,27,28]. For a scoping review of health and medical device development, the work by Matinolli and colleagues can provide valuable insights [29].

### 1.2. Medical Beds

The research subject of this study is the electrical medical bed and its design, whose modern history and related innovations have been explored in recent reviews by Ghersi [30,31]. He identified the electrical beds’ origins with the invention of adjustable sides around 1815 and 1825 [32]. Following technological development, beds gradually transformed themselves into what he defines as Intelligent Mechatronic Beds. In modern electric hospital beds, software and hardware work together, allowing the bed and its components to move concertedly, thus integrating mechanics with electronics and computer science. Nowadays, the advanced versions of these tools usually present an electrical engine moving four different sections. The bed presents three articulated parts (back, thighs or upper leg, calves or lower leg) and a fixed central part. The latter prevents the mattress from deforming and guarantees an equal pressure distribution even if the movement of each section reaches its limit. Moreover, the leg portion of the bed is subdivided into thighs (upper leg) and calves (lower leg) sections. This subdivision allows a slight elevation at the knees level, permitting patients to reach a position similar to an armchair (chair position). Furthermore, modern beds present split side rails, four moving wheels with a brake, control panels for patients and caregivers, removable footboard/headboard and many other features depending on the production company.

**Figure 1:**
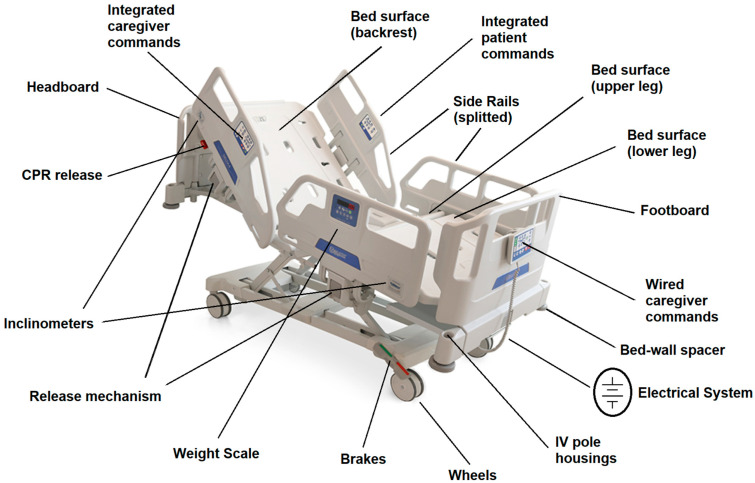
Scheme presenting the structure of a modern electrical medical bed.

A scheme of this structure is shown in Figure 1.

Despite its central role in caregivers’ and patients’ hospital life, the hospital bed has received little attention in HCD research for innovative features. Indeed, it is rare to find examples of complete design processes in the literature. One example is the extensive work conducted by Wiggerman and colleagues [33], where they described the design process starting from an observational study conducted in 29 hospital units in North America. After creating the prototype, they conducted multiple usability tests and concluded their work by listing the selected design features. More common are studies which describe performance tests of new innovative features and their use. For example, some studies tried to detect unchecked patients’ bed exits and fall, since these are severe causes of injuries [34]. Hilbe and colleagues [35] developed a bed-exit alarm system, starting the design process by reviewing the literature and conducting open interviews with 12 nurses. They subsequently built a prototype that has gone through laboratory testing, in which they confirmed its usefulness in preventing falls. Another work by Wolf and colleagues [36] used the same methodology to test another similar system for fall prevention. An important theme is the maneuverability of the beds. A work by Zhou and Wiggerman [37] evaluated the brake pedal location with nine healthcare workers, establishing its design implications and the preferred height for the push handle. Another study by the same authors analysed the effect of two bed features (e.g., Trendelenburg position and maximum mattress inflation) on the caregivers’ physical stress during typical patient repositioning tasks [38]. Some studies have explored the medical bed with subjective methods, for example, by investigating the caregivers’ satisfaction with the hospital bed, highlighting how often the difficulty in maneuvering operations, transportation of patients and bed cleaning are the most physically demanding and troublesome tasks [39]. Another study pointed out the importance of technical support and user-friendliness to influence nurses in using the functions of the bed [40]. Another example is a semi-structured interview study that described bed comfort criteria to create an evaluation checklist [40]. Other examples in the literature describe similar features, but they look at laboratory testing without considering end-users [41,42] or involving novice participants [43,44,45].

### 1.3. Research Objective

As addressed previously, the bed plays a central role in many healthcare environments. It represents the object with which caregivers and patients often interact. In our opinion, it is fundamental that its design encounters end-users needs in order to release part of the work-related stress from people who spend their working time in such stressful environments [46,47]. To the best of our knowledge, the literature lacks comprehensive work describing caregivers’ opinions and needs regarding the medical bed. Therefore, this work aims to provide researchers and companies with a qualitative study that deeply explores this theme. The results will list modern electrical bed features’ advantages and limitations, creating a valuable tool for analyzing bed-related caregivers’ problems and providing guidelines for the future design of medical beds.

## 2. Materials and Methods

### 2.1. Focus Group

This study utilized the Focus Group (FG) technique. This consists of forming a selected group of participants, usually homogeneous (e.g., sharing similar professions, backgrounds and experiences), to enhance their comfort during the discussion of a topic. A moderator is present to propose the questions, manage any problems among the participants, control the time of their interventions and maintain the discussion on the desired topics. Finally, an observer is instructed to pay attention to the non-verbal language of the participants to assist in moderating the debate. Health researchers have extensively used Focus Groups because of their capacity to generate ideas and identify issues [48].

The FG was divided into two distinct parts. The first part of the FG start consisted of a rapid phase of acquaintance with a round of participants’ names, followed by some easy and immediate questions (i.e., When was the last time you used an electrical medical bed? What are the actions that you often perform with the electrical medical bed?). This initial part was useful in breaking the ice among the participants and introducing them to the subject matter. Next, four questions explored the participants’ wishes regarding the hospital bed and its impact on their work. We elaborated four questions starting from the work of Güzelbey Esengün [41] and colleagues, where they subdivided the bed-related arguments into five categories. We excluded economic-related questions because in Italy this is not a caregiver’s responsibility. The four questions were concerned with the impact of the physical characteristics (e.g., height, weight, etc.), the materials used, the electrical functions (e.g., electric inclination of the backrest, lifting of the bed base, etc.), and any psychological feature able to give serenity to the operator. The birth of new ideas on the beds currently in use was stimulated during the discussion to find new proposals and possible adjustments.

Each focus group lasted on average 2.5 h, and was audio and video recorded to permit consequential transcription of the contents. The data analysis was carried out with thematic analysis [49]. The interviews were transcribed starting from the audio recordings. Afterwards, three researchers independently read all the transcriptions, defining and then discussing the emerging themes into which participants ‘answers could be subdivided.

In addition, a series of semi-structured interviews were conducted (6) to deepen the discussion of the topics. The researcher prepared the same list of questions above for FG. The interviews lasted an average of 50 min and were recorded in audio and video. The researchers followed the same analysis procedure as for FG.

### 2.2. Procedure

**Figure 2:**
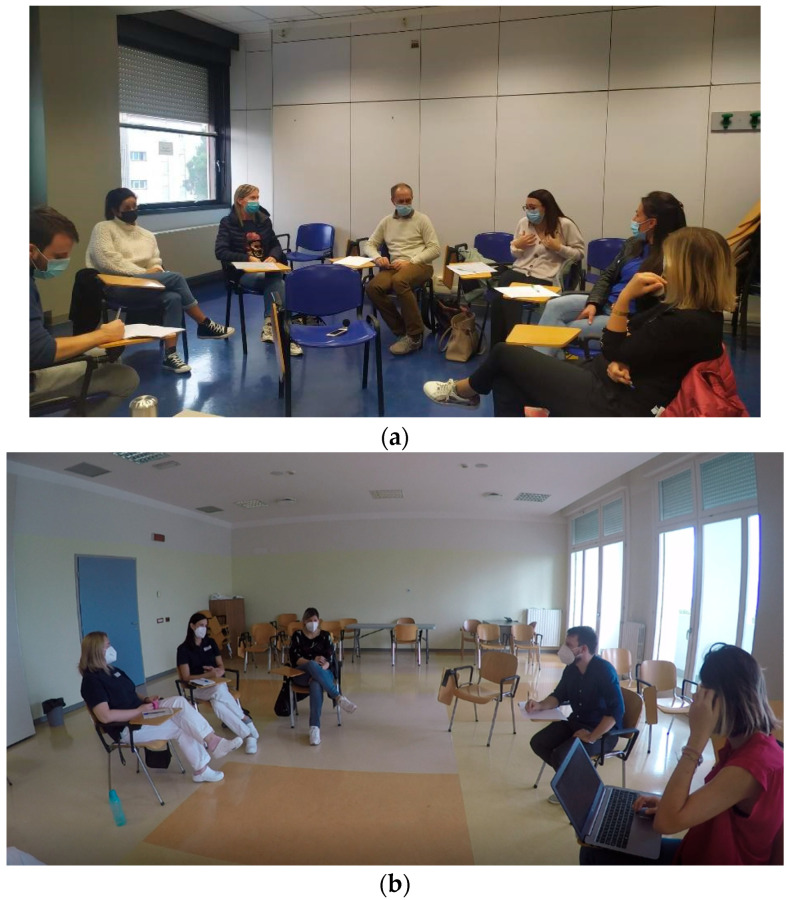
(**a**) Focus Group with nurses employed in a hospital. (**b**) Focus group conducted with a mixed group of nurses, SHO and physiotherapists at a retirement home.

The FGs started by receiving the participants in a welcoming environment, where they could comfortably sit in a circle (Figure 2a,b). The objective was to create a place where participants felt equal and could freely express their ideas. They first completed the informed consent and a demographic questionnaire. Then discussion behaviour rules were listed. Food and water were at the participants’ disposal for the entire duration of the discussion. Once the preparations were finished, the FGs took place.

### 2.3. Participants

All the participants involved in the study were healthcare professionals from different working situations, namely healthcare institutions and hospitals. Daily, these professionals deal with a wide range of patients with different needs and problems. In our opinion, to encourage discussion, it was important that participants in each FGs belonged to the same structured healthcare facility (e.g., institution for the elderly, home care service). Their common experiences could be crucial in underlining their work limitations and criticalities regarding the electronic bed, providing solutions that can be adopted in a wide range of healthcare settings. Consequently, meeting professionals’ different needs could lead to better care for their patients.

To this aim, participants were assigned to six FGs. Specifically, 3FGs collected nurses’ experience (FG1-2) and that of healthcare assistants (FG5) who worked in different hospital wards. Two FGs involved professionals employed in institutions caring for fragile patients (i.e., an institution for the elderly in FG3 and an institution for disabled people in FG4). Finally, in the last FG6, we reported the experience of a group of nurses who provided home care assistance. The whole sample was composed of 29 people (Female = 19, Male = 39, SD = 9). Professionals had an average of 13 years of experience in healthcare (SD = 5.17), and they generally worked with electronic beds daily. However, only three participants reported that they had been properly trained to use the electronic bed during these years.

A brief socio-demographic description of the different cohorts is represented in Table 1.

To further explore the object of the study, we conducted six semi-structured interviews (INT) with bachelor students of nursing science (Female = 3, Mage = 27, SDage = 9.3). They represent the next generations of healthcare professionals, and their opinion could be an opportunity to provide new insight. However, despite already having at least three years of experience with the electronic bed, they were not part of a structured work organization, which was one of the criteria used in designing the FGs. For this reason, their experience was reported separately trough semi-structured interviews.

## 3. Results

During the discussion of the results, we will list every theme that describes a specific element or characteristic of the bed, providing valuable citations. We identify the number of occurrences in which the features appear across the different interviews/focus groups. For each theme presenting comments with more than three occurrences, we provide a graphical representation of the three most common comments and suggestions.

### 3.1. Side Rails

Regarding the physical characteristics of the side rails, the analysis highlights the first element of discussion in their composition (i.e., subdivided into two parts or as a single long side rail).

The participants indicated the multiple side rails as a positive element in 10 occurrences; among the advantages, they can facilitate hygiene procedures for the patient (FG1-P04: “The split ones are comfortable for hygiene”), allow the creation of escape routes for tubes and drains, make restraint less evident (FG-P02: “you can only pull up the side rails of the feet, keeping the head part raised and the patient still feels safer”) and follow the movement of the backrest. However, a negative found for this type of side rail concerns the creation of spaces in which the patient could get stuck. The single side rail was considered as a positive element in six occurrences. Among the advantages of this type emerged the possibility of being lowered with a single gesture (INT-P06: “Just one move to lower it”), preventing any cables from getting stuck or being cut (FG1-P02: “Then in those no wires or drips got stuck”) and removing the risk for the patient of getting stuck between spaces created by multiple side rails (FG1-P03: “With those divided, halfway the space is a danger, they get stuck, it is an escape route”). However, there remains the possibility of the patient getting stuck between the boards that compose the side rail (FG3-P05: “They get stuck in the space between the boards that make up the side wall”).

Regarding their shape, according to the caregivers, they should be curved/rounded (FG6-P05: “They don’t have to be straight, which gives a sense of containment and suffocation, more dynamic”), and lower to reduce the height of a possible fall (INT-P01: “If they are too tall some patients can climb over them and the higher it is, the higher the fall height”). In addition, they should physically support the patient (three occurrences). The participants showed the need to create support points for the movement of patients (FG5-P02: “Good grip helps us to lift them”; INT-P03: “It would be a support for the patient to hang on or sit down, they shouldn’t hang on to the operator”).

Regarding possible functions related to the side rails, some comments (four occurrences) that emerged during the FGs show the need to create an electric height adjustment mechanism (FG3-P03: “Often with anti-decubitus mattresses, they are too low”) or a manual one in case of need (FG4-P03: “I would need a manual mechanism in case of need”). For their release/movement mechanism, it should allow lowering under the bed surface (eight occurrences; FG-P02: “Closing under the support surface”) to avoid accidents (two occurrences; FG1-P04: “When they get off, they cut your feet, giving you a hit”; FG2-P01: “Tall operators bang to maneuver in the center”) and the creation of gaps between the bed and other supports (FG-P01: “Even when they are transported by stretcher/bed or bed/bed, there is a big void”). Furthermore, the mechanism should be easy to use and manageable (three occurrences; FG5-P02: “They should be easy to lift down, even for making the bed”), and equipped with an electric self-locking mechanism (three occurrences) and braked (FG-P03: “Often it can pinch you”). Raising and lowering the side rails should require a fast single action (four occurrences; FG3-P02: “That closes with a single action, which does not become difficult to raise and lower them”), operated with one hand (three occurrences; FG2-P02: “The closure should be one-handed”). Finally, they highlight the need for an alarm for lowered sides (INT-P05: “Maybe you pull up the banks, but you also pull down the sides, in which case you would be notified”).

Caregivers have highlighted how the material that composed the side rails should be light (two occurrences) but resistant (five occurrences). The reasons are safety (FG6-P06: “Often we are alone we have to put a lot of pillows between the person and the edge and they often hit knees etc. creating new injuries”) and comfort (INT-P01: “Patients put hands on them and feel a sensation that is not comfortable, icy or too hot or hard. It could also be therapeutic from a certain point of view, evoking good sensations”). Materials should be soft on the inside and padded (seven occurrences). Furthermore, they should be plastic, fireproof and possibly smooth (two occurrences; FG5-P03: “Smooth would be practical to sanitize”). Furthermore, it was indicated not to use wood as a material due to matters of deterioration and hygiene (three occurrences, FG4-P02: “The wooden sides with a single band, sometimes broke, maybe hitting the lifter. Here we talk more about materials, and some are poor“; INT-P02: “I have seen some with wooden sides, but this is much less hygienic compared to plastic”).

Regarding the psychological impact of the side rails, they have been seen as elements that give safety to the patient and the operator (four occurrences; P05-INT: “Sometimes they give a sense of safety, even for operators”). On the contrary, other participants indicated that these were a limitation to the patient’s freedom since they give a sense of being in a cage (seven occurrences; FG3-P02: “Sometimes they represent a limitation of freedom”). For this last problem, the use of sides without holes (two occurrences; P01-INT: “Very beautiful modern sides, perhaps if they could be full and not angular”) or transparent was proposed. Finally, they proposed avoiding the use of straight bars (FG6-P05: “they don’t have to be straight, which give a sense of containment and suffocate”).

**Figure 3:**
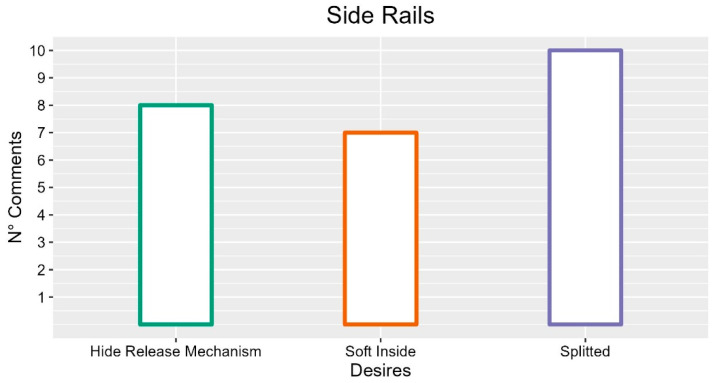
Graphical representation desires with more occurrences for the Side Rails theme.

To summarize, based on comments’ frequency we can say that professionals recruited prefer to work with split side rails, with a soft part inside to avoid patients’ injuries. It seems also important that the release mechanism of the side rails should be hidden. The suggestions with more occurrences are described in Figure 3.

### 3.2. Headboard/Footboard

Regarding the physical features of these components, they should be a low bulky component of the bed (two occurrences; FG1-P06: “It is still a nice piece, heavy, and then cluttered, you no longer know where to put it”). They should also be able to accommodate accessories and shelves of various types (two occurrences; INT-P02: “Headboard/footboard with elements for hanging devices to be used concurrently”).

The footboard and headboard should be removable (four occurrences) for caregivers’ comfort and safety (FG1-P03: “It becomes safer for me too, it’s a convenience”). One comment further proposed that these bed elements could become interchangeable (FG2-P02).

The material of these elements should be light (three occurrences; FG2-P03: “They should be detachable light pieces”) and softer to avoid injury (two occurrences, FG4-P05: “Maybe even a softer material, because they hit their heads”).

From a psychological point of view, these elements of the bed should be quick to detach for the operator (three occurrences; FG6-P03: “I should be able to lift it and quickly access the lower and upper limbs”) and comfortable for hygiene (two comments; INT-P01: “For various needs, orthopedics and machine encumbrance, cleaning even under the mattress”; FG5-P02: “They are easily washable due to removal, they are practical for hygiene”) and for various therapies (FG1 -P03: “We often work from there, it becomes safer for me too, it’s a convenience”).

Therefore, looking at the frequency of comments regarding the headboard/footboard, we can say that the most desired qualities of the bed are removability and quick release, but also lightness of material.

**Figure 4:**
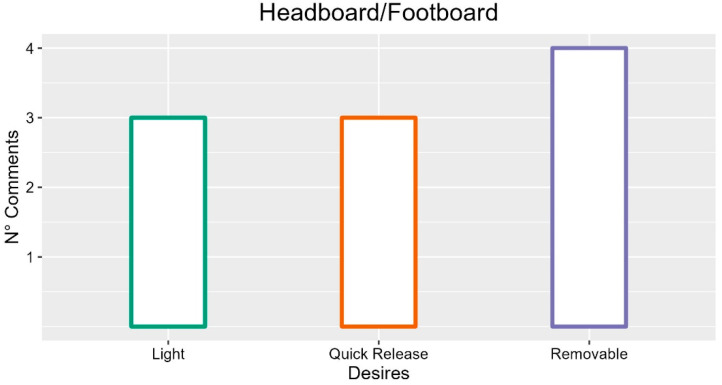
Graphical representation of desires with more occurrences for the Headboard/Footboard theme.

The suggestions with more occurrences are described in Figure 4.

### 3.3. Bed Base Surface

The bed surface, the plan that supports the mattress, has also been mentioned (three occurrences) because it is an element particularly prone to getting dirty (FG6-P03: “It takes liquids of all kinds”). Therefore, the participants would like solid bed surfaces without holes (two occurrences; INT-P01: “All grooves should be covered to facilitate cleaning”; FG4-P02: “Large holes, like normal bed bases, do not allow to put anti-decubitus mattresses”).This has to be waterproof (FG6-P03).

As for the materials, the composition of the bed surface’s cover should be smooth plastic (FG3-P01), resistant (FG6-P02) and fixed (FG6-P05: “Not that it wobbles as soon as you move it”).

### 3.4. Electrical System 

Participants indicated that integrating electric sockets to the bed could be helpful for attaching various instruments (five occurrences) and overcoming issues with cables (five occurrences, FG1-P02: “Often by moving the sides or other, the plugs disconnect”; FG4-P05: “No more cables can be added”).

Regarding the patient, the participants indicated as useful a socket in the internal part of the bed, reachable by the patient, because if placed externally it could be uncomfortable (INT-P04). Moreover, they indicated placing it on the head part of the side rails (two occurrences, FG3-P02: “Then the socket should always be on the head side; instead, they are all on the foot side”). In addition, participants proposed magnetic loading (FG2-P02). The participants suggest integrating more plugs to connect the anti-decubitus mattress (FG2-P02: “when I did orthopedics, if there was an electric bed, you did not have the anti-decubitus mattress because the bed was connected to the plug and then it happened that in the operating room came the ward bed with a different socket and you had to go in search of the adapter, which cannot be used because it is not standard. Then, you have to contact the mattress manufacturer to change the plug”) to attach various electrical tools while moving the bed (three occurrences; INT-P04: “Possibility to use tools even while on the move”) and for USB devices of patients (two comments; INT-P04: “Increased comfort for the patient”).

Regarding the electric cables, the participants expressed the need (two occurrences) to hide them to reduce wear and improve aesthetics (FG4-P02: “The cables have to be hidden, often they are exposed and wear, could be cut”; FG5-P01: “When you move beds they often go under the wheels”; FG4-P02: “Cables should be hidden for aesthetics and patient safety”).

From a functional point of view, the participants highlighted as a practical function the alarm that occurs when the bed electrical plug is disconnected (INT-P03: “Excellent functions, not in all beds but most cases it is the fact that they sound when they are disconnected from the current”).

Furthermore, the autonomy of the bed battery received opposite evaluations. On the one hand, it is perfectly adequate (two occurrences; INT-P01: “Exceptional autonomy”; FG5-P03: “Never been a problem”), and on the other hand, it needs improvements (FG2-P03: “We need more autonomy in travel”). In any case, the autonomy of the battery must be adequate to exploit the bed’s functions also when it is unplugged from the current (INT-P02: “It must have a certain autonomy even when disconnected from the current as its functions are very useful also for example when entering the elevator”). A possible solution to overcome the problem is an external battery to activate when moving the bed (FG2-P01: “It would be nice to have a small auxiliary battery that allows you to be a support to move”).

**Figure 5:**
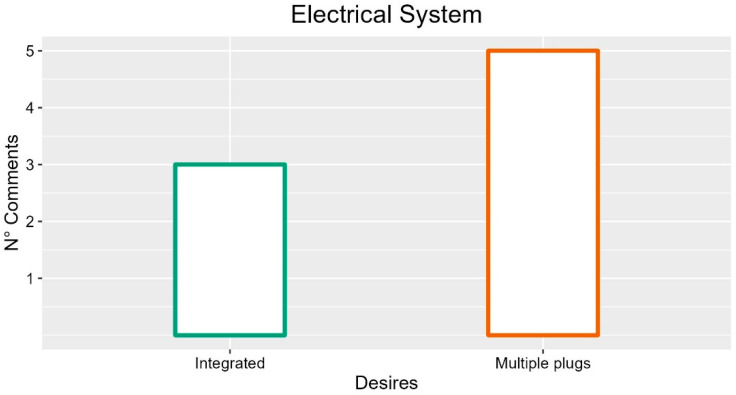
Graphical representation of desires with more occurrences for the Electrical System theme.

So, based on the frequency of participants’ comments the electrical system should be integrated to the bed and should have multiple plugs. The suggestions with more occurrences are described in Figure 5.

### 3.5. Accessories

Although not strictly part of the bed structure, participants often cited accessories as practical elements for their work. In general, the bed should be flexible in supporting the operators and able to accommodate a great variety of accessories to use in various situations (two occurrences; INT-P02: “It must allow the installation of other devices”; INT-P02: “Maybe also that allows you to install applications, devices, such as drip poles, very useful things not so much when the patient is in the ward but during transport, which is a very useful thing”; FG2-P02: “It should be adaptable to many accessories, to reduce the effort of adapting it to different instruments”; FG2-P03: “I also thought that many patients stay in orthopedist ward, and have external instruments. Sometimes using these and making them sit comfortably is difficult. You find yourself in difficulty with back and foot positioning, and sometimes you can’t because the mattress doesn’t allow it. So, personalizing the final part of the bed is important. Because then you need to re-adapt a series of non-functional conditions. Maybe support for accessories on the footboard is missing”).

One of the accessories taken into consideration by the operators is the IV pole, which is considered convenient and useful (four occurrences; INT-P04: “The pole can be a reason for peace of mind because it removes problems with needles and movements with wires attached to the patient”; INT-P05: “Also keeps instruments out of reach of the patient”). For the participants, the bed should integrate the pole, which has to be adaptable (five occurrences, INT-P02: “The IV pole should be foldable, integrated into the bed. Attached to the bed, it would allow it to be used while moving it, and if it were integrated, I would not have to go looking for it”; INT-P04: “Very useful, they must be flexible for different uses or heights...they are not always standard, they should always be present, even in the nursing home bed”; FG2-P02: “The IV pole should be telescopic”; FG5-P02: “It should be easier, more flexible the exchange between IV poles and poles for the triangle”), and resistant (two occurrences; FG5-P01: “They are often fragile, maybe you put the nutrition bags and the stakes because those are heavy”; INT-P04: “Put the nutrition bags, the poles break easily”).

The participants negatively mentioned the hooks for the diuresis bag several times (six occurrences). Their positions are often too low, causing the bag to touch the ground (six occurrences; FG6-P05: “The holder is too low”; FG5-P02: “The attachment of the diuresis bag is not practical, we need a hook to hold it up. Because when you lower the bed, it goes too low for those bags, and they touch the ground”; INT-P04: “It is difficult to find a position where it does not touch the ground and is low enough to operate”). Precisely for this reason, they expressed the desire for a higher or adjustable attack (FG6-P05: “Even the pole they put on beds is sometimes too low, it is so low that you must always remember to pull it up. It has to be adjustable so that I don’t have to pull it up or put the bag on the patient”) and to make it standard equipment (INT-P02).

Furthermore, participants suggested the integration of shelves and various types of storage compartments (four occurrences, INT-P03: “The objects should be inside the figure of the bed, perhaps underneath, so as not to collide with external elements during the transport”; FG4-P05: “It would take a space for the compressor of the anti-decubitus mattress, to store it safely and hidden”, INT-P04: “There could be a basket for patients, for their comfort”; FG5-P02 “Parrot carrier”).

Accessories useful by healthcare personnel are also the anchors for restraint methods (four occurrences; FG1-P02: “It would take a safe and comfortable point to reach. You can’t do it on the edge because you risk breaking his arm”; FG5-P02: “Regarding the hooks for restraint, it would take something more external, more under the bed, something that can be extracted”; FG3-P02: “One thing that is missing is the possibility of putting a restraint belt, at least creating a mechanism to easily hook them. Now the process is very inconvenient”).

The participants also mentioned the mattress and its size, which is often not the same as the bed. Therefore, they reported the need to have mattresses of the right size or to create aids to remove spaces and stop the patient slipping (four occurrences; FG5-P01: “The beds are larger than the mattress, it always goes down. It always has a space where the mattress slides down”; FG3-P02: “The beds and the mattress are sold separately, and for the mattress, which is sometimes wider than the bed, you ruin it with the side rails. You pinch it, it deteriorates”; FG6-P05: “The mattress is small compared to the bed and voids are created”; FG6-P05: “The mattress should have hooks to the bed surface because it often slides down together with the patient”; FG1-P01: “We need a mattress already prepared for bed because changing it and ordering the right ones is a waste of energy, time and funds”);

The triangle pole (two occurrences) is an accessory that the staff rated as uncomfortable (FG5-P02: “The triangle is a bit uncomfortable at times”), heavy (FG1-P03: “Then there is the pole of the triangle which is very heavy. It indeed has to hold the patient, but it is dangerous”) and which needs to physically support the patient’s movement (FG5-P02: “Like the triangle, something to support them to cling to because you cannot leave the triangle there because it is dangerous”).

The headrest (two occurrences) has been cited as a support for the patients’ hair washing (FG-1P05: “Hair washing was not provided, it would take a headrest, like hairdressers. An accessory”) or to support comfort (FG3-P03: “A headrest to support the patient”).

The participants also proposed a bed-wall spacer (two occurrences; FG4-P02: “Something we need to distance the bed from the wall to prevent it from banging, perhaps vertical wheels to slide on the wall”).

Some comments emerged regarding the blanket’s lifter (three occurrences). The participants want it integrated into the bed (three occurrences; INT-P06: “The idea that I could put some kind of extractable structure from the bed that can be composed but that it is already integrated into the bed”) and electrically adjustable (two occurrences; FG6-P02: “The height of the blanket lifter, which is adjustable so that I don’t have to pull it up and put it on the patient”; FG4-P02: “The lift blankets, it’s not a quick thing to take off, you usually leave it there even if you don’t need it. Also, when you make the bed, it’s not nice to look at. So it would be convenient”).

The participants also proposed less bulky bumpers wheels (one occurrence; INT_P02: “They must not clutter up because the operators hit us in agitated moments. Then they must not let the patient feel the impact”).

The participants also find it useful to add an armrest for patients (one occurrence; FG6-P02: “When I have to take a venous route or similar, it would be useful to have a support for the patient’s arm”) and an attachment for the anti-decubitus mattress (one occurrence; FG5-P02: “We use MAD a lot, we have the motor to support. We put it where the large remote control of the bed rests, and it just sits there. Maybe a longer space because they don’t fit together, it’s not very practical”).

Finally, it was highlighted in five comments how the bed-to-bed or bed-to-stretchers spaces cause discomfort among the operators (five occurrences; FG3-P03: “Gaps are created between the bed and prams/stretchers”; INT-P04: “Systems for the interaction between the two are non-existent”). Participants expressed the desire for systems to facilitate this transition of the patient (INT-P04: “It would take a system to be able to move the patient easily”; FG2-P01: “It would take a support base for lateral movement”; FG3-P03: “We need an adjustment in the support surface change”). One of the causes is the space required for the vertical descent of the side rails (FG1-P01: “Even when transported by stretcher/bed or bed/bed, there is a large void”).

Finally, the participants reported that the base of the bed could represent a potential obstacle to the operator’s work (INT-P01: “Often it is an obstacle for patient lifting trolley”).

**Figure 6:**
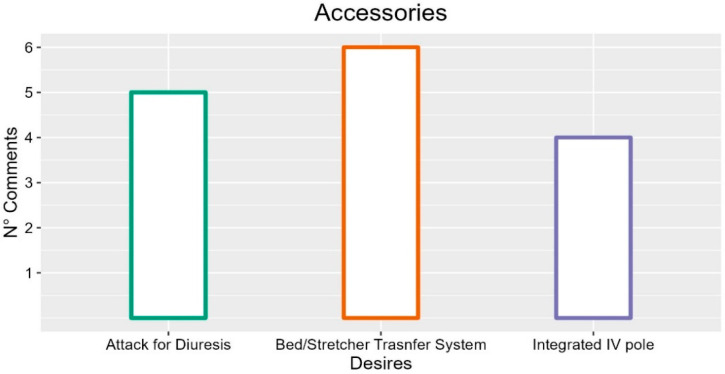
Graphical representation of desires with more occurrences for the Accessories theme.

Therefore, the most desired accessories based on the frequency of participants’ comments are the integrated attack of the diuresis, the bed/stretcher transfer system and an integrated IV pole. Suggestions with more occurrences are described in Figure 6.

### 3.6. Bed Height

The participants reported the height adjustment of the lying surface as a central function for the work and comfort of the operator in nine interviews, underlining several related fundamental aspects. In particular, they highlighted the possibility of setting a minimum height as an element related to patient safety (two occurrences; FG3-P01: “It is essential to reach a minimum (30 cm) for the patient’s height”) and the need to increase the height adjustment range (INT-P04: “It is important perhaps to extend the range in which the bed can actually get up”). Several participants (four occurrences) proposed increasing the speed of the movement in height adjustment (FG6-P02: “A little faster, yes. The whole movement requires a few seconds”). However, some participants suggested that a greater speed could also disorient the patient (two occurrences, FG6-P01: “Many could certainly be disoriented”).

In addition, a participant expressed the desire to have a function to automatically adjust height during dressings (FG6-P01: “I would like a function that when you get to the moment you need to reach out to the patient helps you adjust the height”).

Finally, regarding the psychological aspects related to the patient, the possibility of independently adjusting the height of the sleeping surface is perceived as an element of participation (INT-P05: “Patients could feel more involved if they can change the bed height”).

**Figure 7:**
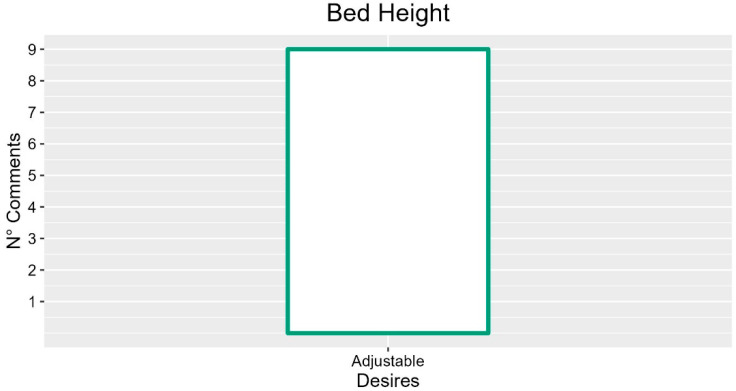
Graphical representation of desires with more occurrences for the Bed Height theme.

The suggestion with most occurrences was the adjustability of the bed height, as described in Figure 7.

### 3.7. Patient Postural Management

Healthcare professionals reported several comments regarding bed functionality to support the patient’s posture.

They indicated the inclinometer because of its usefulness (INT-P02: “It would be useful to make sure that the patient is straight because if, for example, the patient is in Trend, he slips, so I would need an indication when I reach 0°”) and talk about its position, proposing a location on the foot side rail (FG1-P02: “The problem is where it is placed, on the patient’s head it doesn’t help, on the feet it would be much more useful”). However, participants highlighted that a protractor showing the bed and backrest inclinations could avoid the patient slipping at the foot of the bed (INT-P02: “Often, due to inattention, a few degrees down or up is left. In practice, the Trend position does not return perfectly horizontal. It is useful to have the indication of the entire bed plus the backrest to avoid whatever problem”). Moreover, the classic inclinometer, which generally works with a sphere that runs on a lane that indicates the degrees, could present many issues (FG-P02: “30° is not always enough, doctors often ask for particular degrees and to do so I have to see the ball, which fits and is not very smooth”). In addition, participants expressed the need for improvements in inclinometer reliability (FG1-P04). For a better view, a small light has been proposed that indicates the degrees of inclination (FG5-P02: “There is also the small light that tells you how many degrees you stopped”).

Regarding physical elements supporting the adjustment of the patient’s posture, the participants also reported (three occurrences) the need to increase the number of sections (FG3-P04: “Not adaptable to all heights; FG3-P03: “Creation of multiple postures”; FG3-P02: “Cervical section for patient comfort and hypertension prevention”; FG2: P04: “Increase in section number to help the patient find the right position”).

As for the functionality of the different sections, several comments (three occurrences) highlight the usefulness of handling the various parts of the bed. In general, this possibility could reduce fatigue (INT-P02: “I’m more serene because I don’t break my back”) and promote patient independence (FG3-P05: “It certainly promotes self-sufficiency”). Some comments (six occurrences) show that the sections’ movements appear particularly slow, increasing the time spent on activities other than patient care (FG2-P02: “Having them faster for the operator would optimize time and practicality”). Even in this case, however, the participants indicated that these movements should not be too fast or abrupt in order not to disorient the patients (INT-P02: “The movement should be gentle, not rude”, FG5-P02: “Also the speed, but not for the backrest, the movement must have a certain regularity. But to get the bed up it should be faster”).

Staff also reported other features to support patient posture. In particular, the “Trendelenburg” movement was mentioned in seven interviews, four of which highlighted its usefulness as a function (four occurrences; FG5-P02: “It is convenient... even for doctors when the patient is sick”; INT-P01/P04/P05: “Convenient”/”Important”/” Used”). Participants also highlighted that the Trendelenburg’s primary function is bringing patients to the head of the bed with less effort, solving the problem of the patient sliding to the foot of the bed and reducing the fatigue of the staff (FG3- P05: “Generally used to raise the patient”, FG5-P01: “The trend to avoid slipping”). Some participants also expressed the desire for this function to act autonomously (FG6-P02: “I would like the bed to tilt and use gravity to reposition the patient on its own”) or to work even at minimum height (FG1-P04: “A small inclination would be enough without all that height”);

The participants mentioned “lateral tilt” in five interviews, referring to it as a help in repositioning patients to prevent pressure sores or to facilitate the insertion of medical devices (five occurrences; INT-P01: “Move the side parts to prevent decubitus and insert aids”; FG6-P02: “Also to turn the patient on the side, having the bed tilt to one side would help me”; FG5-P03: “It would help me turn them on my side”). The participants also considered important the “Fohler” position. The inclination of the backrest could be particularly useful for allowing patients to eat comfortably (five occurrences; INT-P04: “Useful for eating”; FG5-P02: “Good because if they have back problems, it is useful”). The participants reported its usefulness to support the patient’s exit from the bed (two occurrences; INT-P03: “Support to the patient to stand straight”) and to reach a sitting position for therapeutic and comfort reasons (FG5-P01: “They breathe better, eat better, sit well, comfortable”).

Participants reported functions not yet implemented, such as the possibility of putting the bed in a “standard position” decided with the manufacturer and reachable with a single press on the push-button panel (FG2-P02: “Reset to a position established with the manufacturer”), the electrical moving of the leg part of the bed (FG3-P05: “It could be useful for unloading”) and the scissor opening of the leg section (FG6-P01: “I see a bed that can open as if they were two legs, to help me dress patients’ legs”). A participant proposed a wave movement of the sections for patient repositioning (FG2-P02: “You know I said that the more articulated the better. Think of it divided into ten pieces, with the ability to move like a wave going upwards and then bring the patient up”).

A participant proposed an alarm to remember that a patient needs a change in posture (FG1-P05: “Posture to the right and left I maybe forget, they should be moved after a while, so maybe think of a timer that says you have to move the patient”).

From a psychological point of view, participants highlighted that a bed supporting patients’ movement could reduce their discomfort (INT-P03: “Patient discomfort when the nurse has difficulty in moving him”). In general, the movements of the bed are a factor important to the serenity of the operators (INT-P06: “Then surely having a multifunctional bed which I can therefore manage according to my needs reassures me a lot. To be able to modify the instrument according to the patient’s needs and the patient’s needs that are related to the assistance I have to provide”).

Therefore, participants’ most frequent comments regarding the postural management highlighted the desire of participants for increasing the movement speed while the bed is changing position, but also the necessity of implementing the side tilt and the Fohler position.

**Figure 8:**
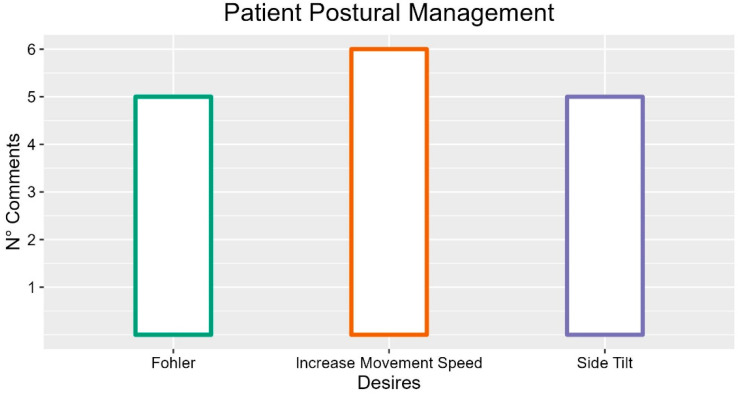
Graphical representation of desires with more occurrences for the Patient Postural Management theme.

Suggestions with more occurrences are described in Figure 8.

### 3.8. Bed Commands

A further topic reported in several comments from the health personnel concerns the commands that allow the adjustment of the bed through a remote push-button panel.

From a physical point of view, a characteristic discussed by the participants concerns its position. The participants indicated the integration in the bank on both sides as the best solution (three occurrences; FG5-P02: “It’s comfortable on the side”; FG1-P04: “You can’t put it on the other side because the cable doesn’t reach it”) to solve the wiring problems, which would tend to get stuck in the mechanisms of the side rails (three comments; FG1-P02: “The wire gets stuck in the mechanisms of the side”). They are also concerned about the frequent falls of the push-button panel due most of the time to the breaking of the hooks (five comments; FG5-P02: “Remote control is most often on the ground”; FG3-P04: “It always breaks and you don’t know where to put it”). In the case of control panels located on the side rails, they highlighted the importance of a blocking function for the patients (four occurrences, FG3-P01: “On the side panel if you can deactivate it for the patient”; INT-P05: “For some particular participants some functions must be blocked, for example those who have lesions at the base of the skull that cannot raise their head more than 30”). A further solution reported by the healthcare staff concerns using magnetic hooks (INT-P01: “the remote control should be out or maybe having some hooks with a magnet”).

The participants also proposed the building of a control panel that integrated other hospital commands (FG1-P02: “I would also unify the remote control of the bed with that of the nurses that usually hangs on the triangle”) and that applies to all beds (FG3-P01).

The buttons should be easy to use (five occurrences; FG5-P03: “Sometimes the remote control locks themselves so it might take a simpler way to lock the keyboard”), intuitive (five occurrences; FG1-P02: “Even overly stylized drawings do not understand them, then they call you to do so”; INT-P05: “Have a guide to read to the patient”; FG1-P06: “They don’t understand that they must first turn on and activate the push-button panel”), reliable (two occurrences FG5-P01: “At the moment they are not very reliable”), easily readable (INT-P05), large, soft (INT-P03) and not too flat (two occurrences, FG1-P02; FG3-P05). Participants reported the importance of constructing buttons resistant to wear (three occurrences; FG5-P01: “Over time they wear out, they break”; FG6-P05: “They erase, they wear out after even only one year”) and to act more quickly (two occurrences).

The healthcare staff then highlighted how the number of the buttons should be less for the patient (three occurrences; FG5-P02: “For example, raising the bed is dangerous for them”) and that a backlight could be useful (two occurrences; INT-P01: “Dim lighting, it must not bother the patient”; FG5-P03: “I would like the lights behind the buttons...I think they go haywire if you leave it on all the time, it must turn on request”). Participants reported a problem with losing grip when using the remote control (three occurrences; FG1-P02: “You can easily lose your grip”). In addition, sound feedback has been proposed when pressing the keys (FG1-P02).

Furthermore, the healthcare staff proposed a wireless keypad (two occurrences), the possibility of controlling the bed with the feet (FG5-P02: “We often have our hands full”) and voice command for hygiene questions (FG5-P02: “the voice command would be beautiful, it would be very helpful in the hospital. Also, because the remote control is dirty, it would also be more hygienic”). Similarly, they proposed a touch screen control panel (FG2-P02: “To avoid wear on the keys and with the recognition of the user who uses it to distinguish operator and patient”) or controlled through applications from a tablet/phone (FG6-P05).

Regarding the materials, the healthcare staff mainly referred to wear resistance, particularly the colours of the icons and the plastics that composed the remote control. They need to be resistant to shocks (three occurrences) and disinfectants (FG6-P05: “Maybe I would add washing instructions”), perhaps thanks to a resistant rubber cover (two occurrences, INT-P06: “The sheath is sometimes severed”). It would also be important that the materials are easily washable.

Regarding the functional properties of the push-button panel, the healthcare staff proposed a distinction between the functions available to the operator and the patient (two occurrences; FG2-P02: “commands should be different between patient and operator”; INT-P01: “Button panel that can be disabled”). The functions should be less for the patient (two comments, FG3-P05: “Patients often find remote controls with many functions that it is difficult to understand”, FG3-P01: “Patients often find remote controls with many functions that struggle to understand”). The participants then addressed the need to save favorite commands (FG2-P01: “Personalization with a badge that I insert and find my favorite functions”) and to activate multiple functions at the same time (three occurrences; FG3 -P02: “I would like to move several parts at the same time with a single key”; FG5-P01: “At the same time they do not go and we must coordinate”). They also highlighted the importance of replacing the remote control quickly if problems arise (FG6-P05: “The remote-control jams from time to time and to replace it you have to detach and reattach the entire bed”).

Psychologically, the remote control helps in the feeling of comfort for the patient (INT-P05: “it helps to feel in control, more involved”) and the operator if unified with the hospital systems (INT-P02: “It is a comfort. However, it should be unified with hospital systems”). A comment also highlighted the importance of understandable icons consistent with those used in their context. Moreover, they found it important to highlight the potential of the hospital bed compared to the domestic one (FG2-P03: “It should be easy to use and that it is understood that it is useful. It must be clear that the bed in your house does not give you these possibilities”). According to a comment, the patient’s use of the remote control could help to involve him/her and decrease the staff’s workload, although some functions would have to be blocked (INT-P05).

In summary, bed commands, if we look at the most frequent comments, should be intuitive and easy to use.

**Figure 9:**
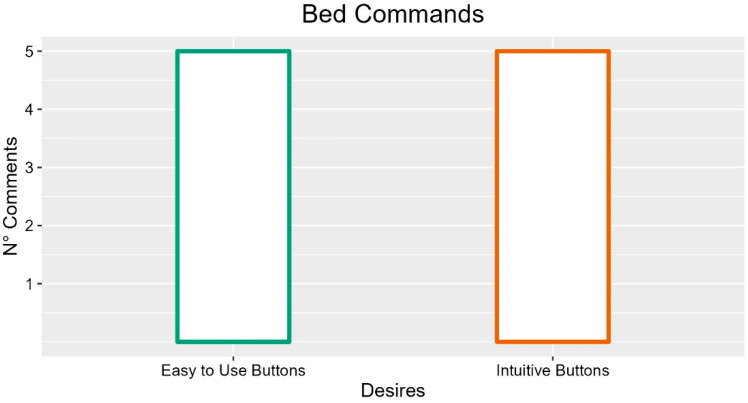
Graphical representation of desires with more occurrences for the Bed Commands theme.

Suggestions with more occurrences are described in Figure 9.

### 3.9. Nurse Call

Users exploited different versions of the nurse call bell. However, they expressed the need to change or modify it (four occurrences). Regarding patients, the addition of a microphone/intercom could allow them to communicate remotely with the ward staff (two occurrences, INT-P04: “With an intercom, to reduce unnecessary interventions or understand where to act first”; FG6-P05: “Intercom type. Maybe the caregiver wakes up as soon as he hears problems”). Furthermore, they indicated the possible addition of a video/monitor (FG1-P05: “Because the doorbells often come off, they are old models”). Regarding its accessibility, the proposals were the implementation of voice command (FG1-P03: “For the elderly, because if they were on the shore, they would not be able”) or bed integrated buttons (FG1-P05).

Regarding caregivers, they reported the need to add a specific urgency alarm on the patient’s bed (FG1-P03: “An emergency alarm, because I always have the bell to call colleagues but if I have an urgency, I have to shout urgency, it will take a bell, a specific sound that for colleagues”). Furthermore, the doorbell was seen as an element capable of creating tranquility in the nurse’s work (INT-P04 “the doorbell takes away a bit of anxiety”). In summary, the participants most frequent comment regarding the nurse call bell highlighted their dissatisfaction with those they have in their workplaces.

### 3.10. Patient Parameters

Several comments from operators highlighted the need to monitor patient parameters through sensors integrated into the bed (five occurrences, FG1-P01: “Parameter detector incorporated in the bed”), and display data in an integrated monitor (four comments, INT-P04: “Monitor with data and vital parameters of the person”, INT-P05: “If there was a way to integrate also a monitor, ECG, breathing, waking state”).

Another tool mentioned by several caregivers was the weighing system, which was indicated as fundamental in two interviews (“FG1-P05: The weighing system is essential because you don’t have to lift patients to weigh them. I had patients weighing 180 kilos”; FG5-P01: “Doctors always ask for weight. Even for therapies, and I don’t want to have to use the lifter”). The proposal for a catheter weighing system was also mentioned for its possible usefulness (three occurrences; INT-P03: “I was thinking, for example, that the catheter was maybe attached to something, to a bed sensor so that the read can directly record even more precisely”; FG1-P05: “Then I come from a reality where dialysis is needed”).

Finally, the parameter detection for patients would also allow the implementation of alarms considered useful by the operators. For example, they indicated the bed alarm for patients’ exit (four occurrences, FG5-P02: “A bell when they put their feet out, a sensor connected to the bed that says there are particular movements”, INT-P05: “Many beds have alarms when they hear the patient get up”) and for agitated patients (one occurrence, FG5-P02: “or maybe agitated”).

**Figure 10:**
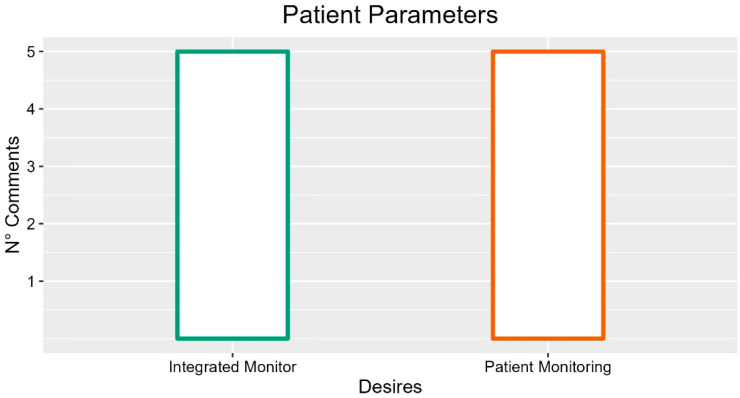
Graphical representation of desires with more occurrences for the Patient Parameters theme.

In summary, suggestions with more occurrences were the presence of patient monitoring and an integrated monitor, as described in Figure 10.

### 3.11. Bed Size 

Regarding the bed size, the main comments from operators concerned length and width. In particular, the participants expressed the desire to have extendable beds to adapt to patients (eight occurrences; FG3-P03: “The bed must be extendable, also because the average height is higher than in the past”; INT-P05: “Bed extension must be there to have more space for tall patients”; FG1-P02: “There must be, but the mattress slips and is an inconvenience”), and to have wider beds (four occurrences). The main reason was increased patient comfort (four occurrences; FG3-P05: “We often have overweight patients”; FG2-P01: “For people accustomed to two squares which will have to stay a long time”). Furthermore, as regards the bed’s width, a participant proposed the possibility of making it adaptable to the patient (FG1-P03: “We would need a bed spreader. If I were in them, I would have claustrophobia”). Some have instead pointed out that the bed should be slightly narrower to facilitate movement across the doors (two occurrences; FG2-P04: “Reduction in width for door passage”; FG5.P02: “Often the rooms are small, and the bed is bulky”).

**Figure 11:**
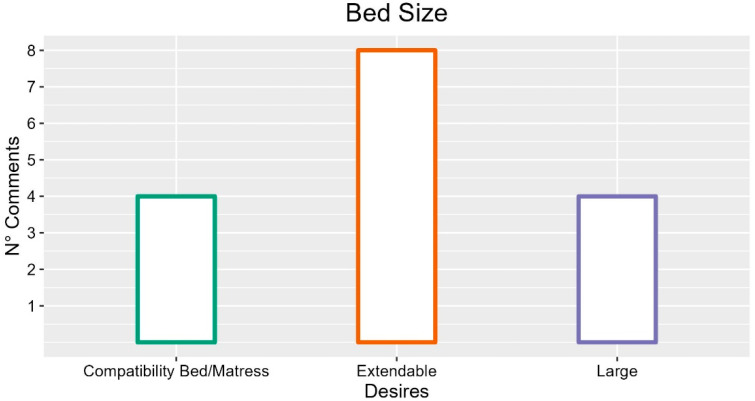
Graphical representation of desires with more occurrences for the Bed Size theme.

In summary, the most frequent comments regarding bed size and the suggestions with more occurrences are described in Figure 11.

### 3.12. Bed Weight

Several participants reported that the bed should be as light as possible (eight occurrences; INT-P05: “It must not be excessive, often the structure is very heavy”; FG2-P03: “Usually two people have to move”; FG5-P03: “It should be light, for maneuverability more than anything else”), especially as regards the removable components (one occurrence, FG2-P03: “The single piece that I have to change in the case must be the light one. I have to be able to remove pieces such as back or footboard with ease, must be light”).

On the contrary, some comments underlined the importance of the weight of the bed (four occurrences, FG1-P05: “Electric beds should weigh, for me it is fundamental”), especially as regards the possibility of moving patients (one occurrence; FG1-P05: “Weight is fundamental because if the bed weights it helps moving patients”).

Finally, some of the participants did not consider the weight of the bed relevant if the maneuverability is not compromised (two occurrences; FG3-P02: “If there are good wheels, it doesn’t matter”, INT-P05: “Simple maintenance of the wheels could help”).

**Figure 12:**
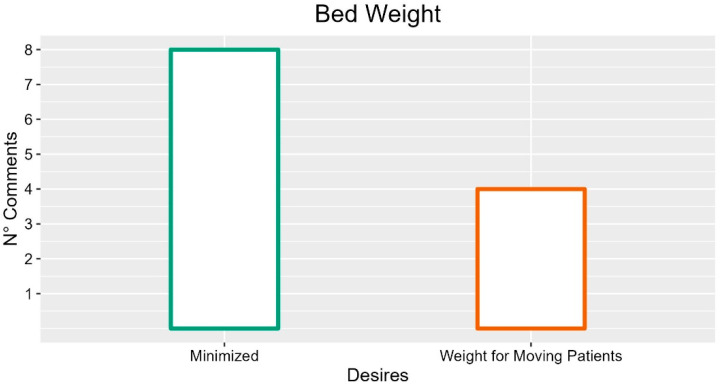
Graphical representation of desires with more occurrences for the Bed Weight theme.

Suggestions with more occurrences are described in Figure 12. The frequency of comments highlighted that the bed weight should be minimized for the participants, even though it has been also suggested that the weight of the bed is an important feature for moving the patients around while on the bed.

### 3.13. Manoeuvrability

Participants’ comments regarded both the brakes and the wheels of the bed.

The participants highlighted that brakes were adequate (three occurrences; INT-P01: “That’s okay”). Furthermore, they needed to lock all the wheels with a single brake (two occurrences; FG4-P02: “single block for all wheels”) located in an accessible spot.

Several comments on wheels highlighted the need for high maneuverability (six occurrences; INT-P02: “They must be more maneuverable”; FG2-P02: “The beds are difficult to move”; FG1-P05: “They cannot be maneuvered alone”; FG5-P03: “My ward is narrow, they are difficult to move”).

Participants then highlighted problems and some possible improvements. For example, they are subjected to wear (FG2-P01). The main problems, according to the comments, concerned the movement of the bed, similar to a shopping cart (two occurrences, INT-P05: “wheels like shopping carts”), and difficulties in turning (FG2-P02: “go straight when cornering”). Participants then proposed multiple driving modes (FG3-P01: “Four free wheels or with the two fronts locked to face curves or straights”) and retractable wheels (two occurrences; FG4-P02: “They would make the minimum height lower”; FG3-P01: “They would promote the appearance of the bed in that of a house”). A further proposal concerns the presence of a fifth motorized wheel (two occurrences; INT-P03: “The weight is often excessive”) and the integration of shockproof materials (INT-P02: “the wheels must be fully functional, the material has to absorb the shocks for when I skid so the patient does not have the feeling of having an accident”).

The physical characteristics that seem to have the greatest impact on the maneuverability of the bed relate to its size and weight. Comments about these elements can be founded in the respective paragraphs.

**Figure 13:**
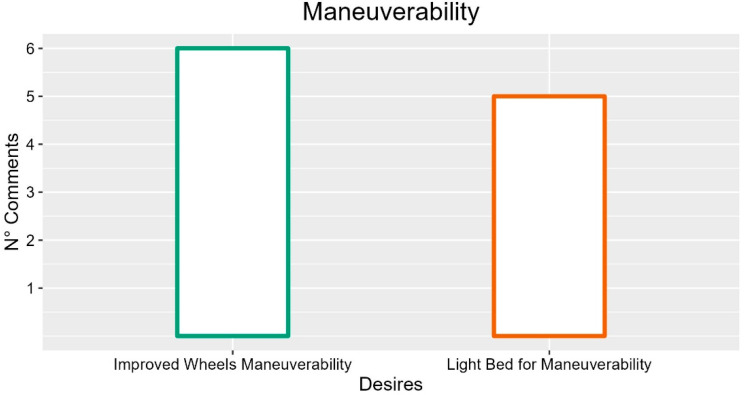
Graphical representation of desires with more occurrences for the Maneuverability theme.

In summary, the most commonly desired features for the electric bed movement concern improving wheels’ maneuverability and reducing the bed weight (this feature is according to what has been highlighted in Section 3.12 about bed weight). Suggestions with more occurrences are described in Figure 13.

### 3.14. Materials

Participants cited many general characteristics of the materials that should make up the bed. According to them, these should be smooth (four occurrences), robust (seven occurrences) and easy to clean (10 occurrences).

The material most suggested by healthcare personnel is plastic (four occurrences) rather than metal, as it is antistatic (INT-P05: “in plastic, for an antistatic issue, if an emergency happens and you have to defibrillate, and you made it in iron, you risk the propagation of the impulse for the patient attached to the bar with his hand. Obviously, if it conducts electricity, you may not have noticed it, but you are touching the bar, you also take it too”), a more welcoming element (INT-P05), more comfortable to the touch (INT-P03: “Plastic materials rather than ferrous, iron is cold”) and does not produce an anxious sound (INT-P04: “Iron, creak, paint that goes away fuel anxiety”).

Another material that the healthcare staff has advised against is wood (four occurrences), both from a hygienic point of view (INT-P02: “Wood finishes: less hygienic than plastic, it is damaged more and more aggressive products must be used to sanitize”) and because of its fragility (four occurrences, INT-P05: “The maintenance that must then be done in the wooden ones is absurd because obviously, the wood ruined fast, the wood breaks”). One comment proposed using plastic-coated metal materials as a solution, creating a robust and antistatic structure (INT-P05: “Maybe you know it is easier for plastic to break than iron, but maybe an iron covered with plastic would be it would be better”).

The colours should be resistant to wear both for aesthetic and patient safety issues (five occurrences; INT-P02: “They must resist maintenance and wear”; FG1-P02: “They must also resist gastric materials, sometimes we have nasogastric tubes and acidic material comes out and the stain remains even if you wash with bleach”; FG2-P02: “Plastic with single paste color, resistant to scratches”; FG6-P01: “That the color does not melt in the heat maybe and that they cannot release toxic substances, resistant to disinfectants”).

As far as the shape is concerned, the comments of the healthcare staff showed that it is necessary to minimize the edges and cracks for hygiene (four occurrences) and make sure the structure is composed of a reduced number of elements (INT-P04: “Few to clean easily and prevent infections”) which, if covered by a covering (two occurrences) or removable, would facilitate their sanitation (three occurrences). Furthermore, the shape of the bed should be subordinated to its functions (INT-P02: “Not essential but must be subordinated to functions”).

**Figure 14:**
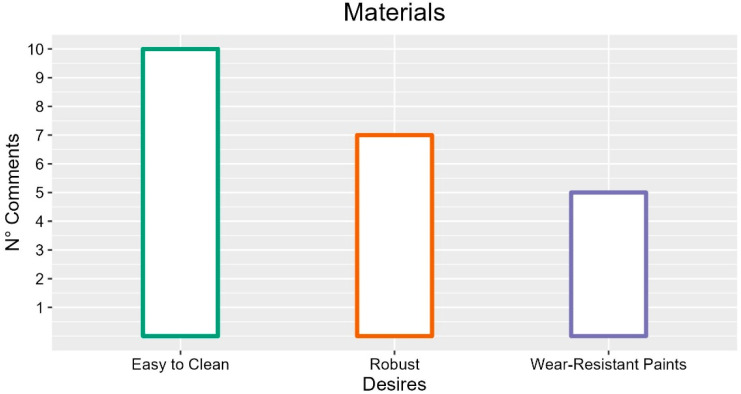
Graphical representation of desires with more occurrences for the Materials theme.

The most cited qualities of the electric bed highlighted the participants desire for easy to clean and robust materials with wear-resistant paint, as described in Figure 14.

### 3.15. Maintenance

Regarding bed maintenance and assistance in case of malfunctioning, several comments concerned the need to have manual control of the electrical movements. In case of broken electrical engines or commands, caregivers must not lose control of the bed (nine occurrences; FG1-P05: “Maybe foresee that if something breaks there would be a manual mechanism, an emergency lever”, FG4-P03: “Manuals even in case of problems”, INT-P01: “Like the CPR lever, to get up quickly without remote control”). Furthermore, the participants suggested the possibility of removing the single defective elements to be repaired or replaced without having to stop the use of the entire bed (three occurrences; FG2-P02: “Interchangeable, which if a piece breaks, you detach it and change it immediately”).

Regarding the implementation of functions related to bed maintenance, the participants proposed a sensor that detects failures (one occurrence; FG1-P05: “When the bed breaks, it is not possible to think of something, a sensor, which signals the problem?”) and an automatic alarm for assistance in case of failures (one occurrence; FG1-P02: “There could be something that gets the signal to the company and they know they have to do this without making emails, requests, etc.”). To summarize, the most frequent comment highlighted that a manual control should be present in case of malfunction of the electrical system.

### 3.16. Aesthetics

Several participants mentioned aesthetics as a fundamental element for patient comfort. In particular, a central aspect concerned the possibility of making the appearance of the bed less hospital-like and more similar to a domestic bed (four occurrences; FG2-P03: “You must also make it beautiful to the eye, with something familiar, which leads back to the domestic context, remaining in a hospital context”). In particular, according to the participants, the appearance of the bed should be modern (one occurrence; FG3-P03: “Positive feeling due to being on a technological object for comfort”) and with a rounded structure (1 occurrence; INT-P01: “Make the surfaces a bit like to say rounded and smooth a bit everywhere”). Participants also addressed the possibility of hiding some elements, such as mechanical parts (one occurrence; FG3-P02: “Then also cover the mechanism of the bed, it should not be visible”) and wheels (one occurrence; FG3-P01: “Even retractable wheels do a lot, you have a bed with four legs”). In addition, four participants proposed colored or oddly shaped beds for children (four occurrences; FG2-P02: “For children full of drawings, possibly even with strange shapes just the bed”, FG6-P05: “We can also have children as patients, giving them a colored one would be more cheerful”).

Regarding the color of the bed, most of the participants proposed avoiding brilliant colors and choosing instead warm and relaxing ones (eight occurrences; INT-P04: “They create a welcoming environment, they must be simple and relaxing, make you feel like at home”; FG6-P01: “Maybe pastel colours”, INT-P03: “If not too strong they can help to give serenity”) or shades (one occurrence; INT-P05: “A little more nuance that at least, I’m not saying it makes you feel at home (...) but at least you look at yourself in your bed and have this feeling of welcome for a moment”). Finally, participants underlined the importance of choosing a color that complements the appearance of the room (two occurrences; FG6-P03: “That fits well into the room”, FG4-P05: “The top would be the bed in the same color as the wall”). Many participants indicated fake wood as the color that better simulates a domestic environment (six occurrences; INT-P04: “Making them like wood to give a sense of home, clean and tidy”). Some participants also suggested avoiding white because it is more prone to getting dirty/stained (two occurrences, FG6-P02: “I would avoid white because it gets dirty”, FG2-P04: “Even the color of the bed may not be white”).

**Figure 15:**
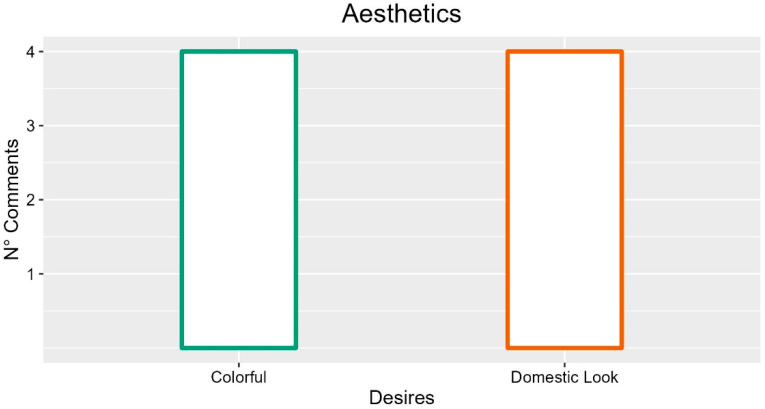
Graphical representation of desires with more occurrences in for the Aesthetics theme.

In summary, the most frequent comments about the electrical bed aesthetic highlighted that it should be colorful but also have a domestic look. Suggestions with more occurrences are described in Figure 15.

### 3.17. Lights 

The participants proposed several lighting systems for integration into the bed. The first was a soft night light proposed for patients’ well-being and to support caregivers’ work (five occurrences). They also proposed a courtesy light to support the work of the operators (two occurrences; INT-P05: “A light would be useful because it often bangs on the bed at night”; FG6-P04: “We often call the caregiver with a mobile phone but having a mobile light would be better, perhaps with a flexible rod. Also, for blood sampling”) and a system to illuminate catheters or bags (two occurrences; INT-P01: “Lights aimed at the catheters to identify their position”; FG6-P03: “A light under the bed to see the state of the urine bag”).

For patients, participants proposed an external courtesy light for getting out of bed (three occurrences; INT-P01: “Lights directed downwards, to reduce ambient light when they have to go out) or an internal one (five occurrences; INT-P03: “An adjustable reading light”; INT-P05: “An adjustable and customizable light would make them more involved”). The latter could make the environment more domestic and welcoming for the patient (two occurrences; INT-P05: “Little things that make them feel, I don’t say at home because obviously, patients will never be able to feel at home, but at least a little more comfortable”). However, several comments underlined the importance of being able to adjust or keep the intensity of the lighting low, to avoid disturbance or discomfort to other patients (four occurrences; INT-P04: “That it is more adjustable because sometimes they make a light and there are patients who want to sleep with light, patients who want to sleep without light”).

**Figure 16:**
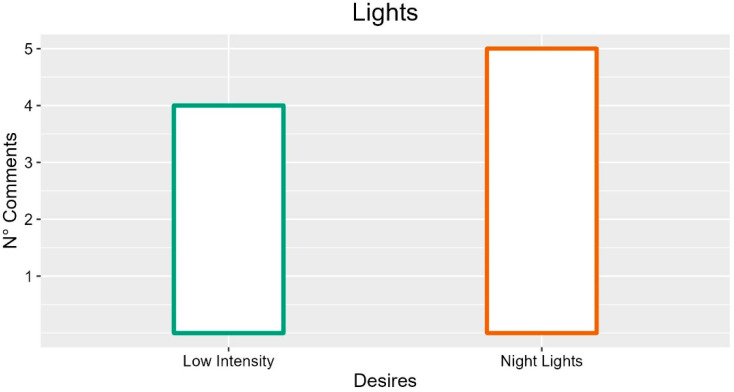
Graphical representation of desires with more occurrences for the Lights theme.

In summary, the suggestions with more occurrences are for integration of night lights and that lights should be at low intensity, as described in Figure 16.

### 3.18. Patient Relaxation 

Finally, the participants proposed accessories/functions to promote the patient’s well-being and relaxation. Firstly, they proposed the installation of a screen for video calls (FG5-P02: “A small screen where they can see something or make video calls”). Moreover, they proposed the addition of a surface for putting a television on the bed (FG4-P02: “A TV stand that can be raised/lowered, perhaps at the foot of the bed”).

In addition, the participants suggested the implementation of an audio system (four occurrences; FG5-P02: “Having a relaxing music on the bed, already inserted in it could perhaps relax them. Maybe something background, personal for not annoy others in the room”, FG6-P04: “Also an integrated radio”, FG6-P03: “Bluetooth connection with two speakers”), and an intercom system for children (one occurrence; FG6-P05: “An intercom-type microphone for children so I hear that it happens even if I sleep in another room. Maybe the caregiver wakes up as soon as he hears problems”).

**Figure 17:**
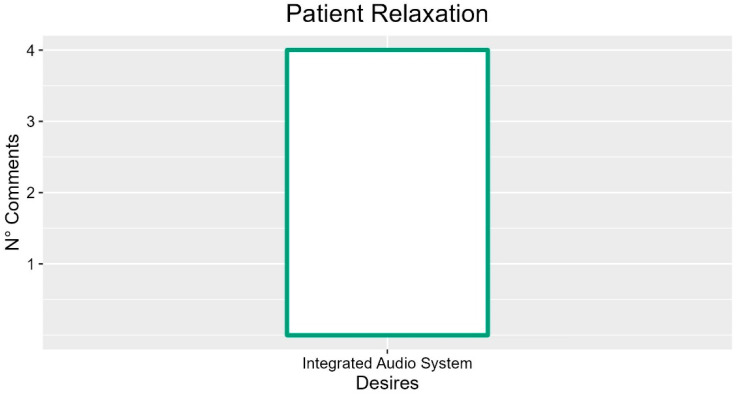
Graphical representation of desire with more occurrences for the Patient Relaxation theme.

The suggestion with most occurrences for an integrated audio system for patient relaxation. as described in Figure 17.

## 4. Discussion

**Figure 18:**
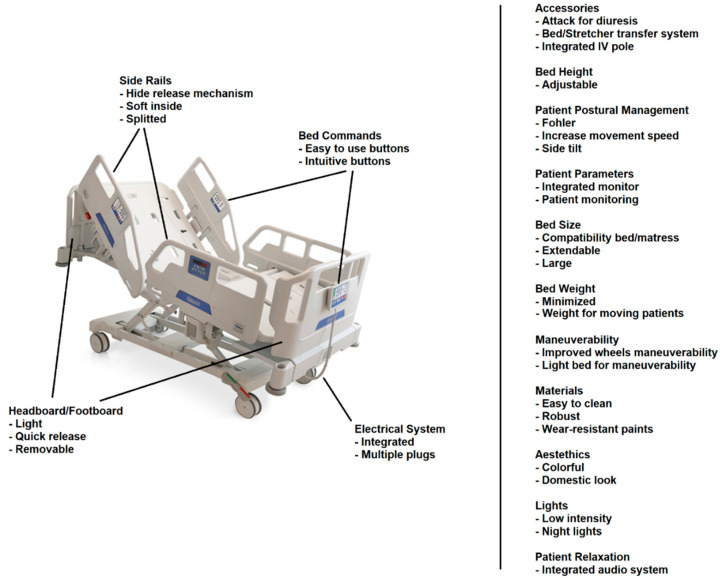
Graphical summary of the results of the study.

The primary aim of this work was to research end-users’ needs regarding hospital medical beds while considering these as a working tool. Indeed, we focused on caregivers’ opinions, including nurses, nursing students, social-health operators and physiotherapists. Moreover, for a comprehensive vision, we involved people from different healthcare realities, such as hospitals, retirement homes for the elderly, retirement homes for people with disabilities and domiciliary assistance. The results present an extensive overview of almost every element of the hospital bed, listing limitations, strengths and caregivers’ necessities. A graphical summary of the results is presented in Figure 18. The complete list of caregivers’ desires and the relative number of comments is provided in Appendix A.

This work’s design suggestions can help future bed designers begin their product development from an advanced starting point, adding necessary adaptations relevant to the application environment. Moreover, new bed designs based on this work will have the advantage of considering users’ opinions. To the best of our knowledge, the literature has almost ignored these themes regarding medical beds, since company design is traditionally a top-down or secret process.

Our work, therefore, identifies some of the main design challenges that can be faced thanks to these results.

### 4.1. Design for Physical Workload Reduction

The first challenge is the reduction of workload. Research approached this problem extensively, especially among nurses, addressing it as a cause of low back pain [50], burnout [51], performance reduction [52], quality of life [53] and many other factors. A recent study also found that the number of steps and time pressure represent possible operational causes of high workload [54]. Therefore, our results could help reduce the time spent on procedures, reducing the number of actions needed to perform a procedure or making it faster. For example, our study addresses the importance for designers of researching the best movement speed for adjusting bed height and sections, finding a compromise between safety and time-saving. Moreover, our participants addressed the importance of decreasing the commands’ response time.

Many suggestions (i.e., easy-to-use side rails, light headboard, etc.) indicate a great involvement of bed components in increasing physical fatigue, which can be reduced by designing them more lightweight and more usable. Specifically, our participants address the need for lighter and more usable side rails, headboard/footboard, and less complex button panels. This finding is supported by the literature, which addresses the importance of ease-of-use [55], design for workload reduction [56], and reduction in complexity of bed commands [57] in healthcare environments. In particular, the headboard/footboard should be easily and quickly removable.

The maneuverability of the bed is another theme that can influence caregivers’ physical workload. Many comments addressed the importance of facilitating the beds’ movements. For example, lightening the bed, providing multiple driving modalities (i.e., blocking four or only two wheels), and adding a fifth motorized wheel can help caregivers reduce fatigue in moving the bed to and from different places. Among these suggestions, the fifth wheel is the only one explored by the literature [5,58].

### 4.2. Design for Bed Adaptability to Different Situations

Designers should pay particular attention to increasing caregivers’ agility during multi-tasking activities frequent in the healthcare environment [59]. In this case, technology should help develop more flexible and adaptable instruments. In our study, participants highlighted that accessories and electrical systems (e.g., standard and magnetic plugs, USB ports) should be integrated into the bed structure (headboard/footboard can be a valid support for these accessories), permitting their use in every situation. Our study also provides many suggestions for accessory improvements, such as an attachment for diuresis bags, integrated blanket lifters, a system for the patient transition from bed to stretcher, and a light for catheter bags. Some of these are already the subject of research. For example, systems for transferring patients from the bed to other supports can reduce physical stress [60]. Our study can provide designers with suggestions about what kind of accessories caregivers want to be directly integrated into the bed.

Many other bed components have been highlighted as too rigid. Participants indicated that side rails design should include systems to adjust the height and the importance of the standard raising/lowering system for bed height. Moreover, another flexibility issue regards the bed battery, whose duration seems too short when the bed is not linked to an electrical plug. Consulting the results for brakes, bed commands, section number, inclinometer and bed base, designers can find suggestions for more flexible bed components. Among these, only bed commands [57] and brakes [44] have been researched in the literature, but not for flexibility reasons.

To the best of our knowledge, the literature is scarce on the importance of technology flexibility and efficiency of use in the healthcare environment.

### 4.3. Design for Patient Safety

Another critical theme highlighted by our participants is patient safety. Some of the elements cited in this study represent known problems in the healthcare environment, for example, the safety of the side rails [61,62,63], instrument maintenance [64], patient monitoring [65,66], or falls from the bed [67]. Regarding the side rails, participants addressed the need to design the internal part with a soft, smooth material to give them an appearance that can avoid the patients’ feeling of being imprisoned. Other pieces of advice represent features that can be found in modern hospital beds.

Our study also presents an overview of caregivers’ desire regarding hypothetical smart beds. Many suggestions represent systems explored by literature or industry that are still not usually implemented in standard beds. These are indications about the state of side rails state [68], posture alarms [69,70], video monitoring [71,72], patient vitals parameter monitoring [73,74], bed exits [75,76] and agitated patients [77,78]. A few new suggestions from our study are a system that can automatically reposition patients through a wave movement of the section, and automatic detection of bed malfunctions, followed by an alarm to the company assistance department. The users’ discussion demonstrates caregivers’ great attention to the problem of patient positioning, a significant issue linked to bed sores [79]. This study presents a comprehensive vision of what functions caregivers imagine in a smart bed, helping designers select what to implement in their products.

Moreover, the results show caregivers’ attention to particular bed positions and movements, such as lateral tilt, Fowler and Trendelenburg. This study shows that designers and companies should provide these with the standard version of their beds.

We believe that these findings can help designers increase patient safety.

### 4.4. Design for Easy-to-Clean Bed

Participants addressed some bed elements as especially subjected to dirtiness. Indeed, following the study results, designers should care about ease of cleaning for bed base surfaces and materials used in building the bed and side rails, since environmental hygiene represents a critical issue in healthcare environments [80,81]. The literature has poorly explored this argument, focusing on the general study of hospital materials [82] or other technologies (e.g., wearables [83]). Still, our study shows that caregivers involved in bed cleaning daily have given this problem great attention.

### 4.5. Design for Aesthetics and Durability

The results of this study highlight the importance of the materials used in bed development, particularly the importance of balancing durability and appearance. Regarding the former, many comments highlighted the robustness of materials as an imperative characteristic since medical bed components (i.e., control panels, buttons, wheels and materials in general) are usually subject to breakage and wear. Participants indicated mainly plastic materials for the external component of the bed and metallic ones for the base.

In parallel, the visible components of the bed should be aesthetically appealing. The discussion of colors and exposed electrical cables highlights that caregivers desire medical beds with a more home-like appearance. This problem of medical bed design has been explored poorly, with few studies focusing on its appearance and only for elderly retirement facilities [84] or homestays [85], representing a new research argument in the field. Some comments pointed out the importance of hiding aesthetically unpleasant parts, such as wheels and electrical cables. As in many other design fields [86], reducing elements and increasing appearance simplicity could help design more appealing beds.

### 4.6. Design for Patient Comfort

Finally, our participants show caregivers’ worries about patient comfort, suggesting bed improvements for this purpose. Their suggestions were predominantly regarding different types of lights (i.e., for bed exit or reading) and for entertainment (i.e., screen for video calls, TV stand, audio system, intercom). The side rail has also been addressed as a possible source of help for patients, providing support when they need to leave the bed. For the components in contact with the patients, caregivers also address the need to create soft or smooth surfaces, including control panels. Indeed, our study shows that designers need to take care of caregivers’ interest in providing a comfortable stay for patients [87,88] and the importance of usable push-button panels [89]. The latter should be easy to grip and provide appropriate feedback, as the literature suggests for other technological devices [90,91,92].

### 4.7. UX Design of Medical Beds

This work highlights the characteristics the medical bed should or should not have in the caregivers’ opinions. In this case, the primary concern of users was the creation of good equipment for all the categories listed in the Results section. Indeed, by applying such design guidelines, it will be possible to create tools to help people achieve their goals with minimal effort [93], contemporarily improving their performance [94]. Therefore, improving the user experience (UX) and usability of electrical medical beds is fundamental. Despite these two constructs showing differences in concept, meaning and utility, usability has often been indicated as a part of the user experience [95], especially for medical instruments, which are also poorly explored in the literature [96]. According to the work of Bitikina et al. [96], usability and UX are defined by the International Organization of Standardization (ISO 9241-210, 2010). UX describes the users’ response and perception of the use of a product, while usability concerns the capacity of users to achieve goals with efficacy, effectiveness and satisfaction in a specific context. We believe that the present work could significantly contribute to our knowledge of UX applied to the medical bed context. Therefore, we selected from the results some themes participants discussed that could be reconducted into these fundamental concepts.

#### 4.7.1. Emerging Factors for User Experience

The FGs highlight multiple factors that could contribute to developing electrical medical beds that provide a good User Experience. In Table 2, the UX/Usability constructs that emerged from our FGs and previous studies that have driven our guidelines are reported. From the requirements underlined by the participants, these UX/Usability categories emerged related to electronic beds.

Follow the UX constructs that emerged from the participants’ requirements, which could provide useful guidelines.

##### Safety

Safety. The caregivers indicated that the bed could provide a safer environment for them and their patients.

Reliability. Lastly, the bed itself should be a reliable tool. Indeed, many participants addressed problems of robustness, deterioration and the necessity for good support from the production company.

##### Comfort

Another essential feature of the medical bed is comfort. In this case, the meaning of this feature changes across the considered users. For patients, the bed and its accessories are tools to provide a comfortable environment and ameliorate the quality of the stay.

For the operators, instead, it provides a comfortable working tool and represents an instrument that could help them with their everyday work.

##### Ease of Use

Strictly connected to this theme is the next feature, ease of use. In this case, the operators cited this factor in many bed-connected procedures or elements that emerged during the FGs (e.g., for the transition of patients from bed to other carriers, or the control panel’s buttons).

Ease of Cleaning. One of the most frequent actions during the caregivers’ work is cleaning the bed. For this reason, participants indicate ease of cleaning as a fundamental feature for many parts of the bed (e.g., control panel) and for the composed materials.

Space Saving. The participants showed the importance of having removable parts that create as little encumbrance as possible.

Intuitiveness. Participants mainly cited this characteristic for the bed commands, where they highlighted problems, especially for patients, in understanding control panel icons.

##### Timesaving and Workload Reduction

Timesaving. Another important theme is the reduction of wasted time. Many bed features permit the saving of caregivers’ time, allowing them to perform procedures faster.

Workload Reduction. At the same time, a well-designed bed could be a helpful ally in reducing the physical workload and for problems such as back pain.

Maneuverability. The bed should be highly maneuverable to reduce time consumption during ward movements and possible collisions.

##### Perceived Usefulness

Perceived Usefulness. The participants often describe some bed elements as useful, indicating perceived usefulness as an important factor in the overall experience.

Usefulness being a helpful element, the bed could become an instrument that could give serenity to caregivers.

##### Flexibility

Flexibility. Linked to the last aspect is also the importance of the high flexibility of the bed and its accessories. The participants highlight that the bed should be adaptable to different situations and procedures, giving so the possibility to be a good help for every situation.

##### Aesthetics

Regarding this last point, the participants addressed this aspect of the bed as an essential element. The bed’s aesthetic could lighten the oppressive environment of hospitals or retirement homes for patients.

Home Appearance. Many participants highlighted that the medical bed should be more like a home bed, allowing patients to be distracted from their conditions and experience a cozier hospitalization.

Patients’ Serenity. A good-looking bed could have its role in providing serenity to patients.

## 5. Conclusions

This work aimed to exploit HCD methods to identify the caregivers’ needs regarding the electrical medical bed. Through a thematic analysis of six focus groups and six interviews, we identify 17 themes representing an equal number of characteristics/features that this tool should present for our participants. Furthermore, our work suggests some user experience guidelines that could help to create more usable and enjoyable instruments. The strength of this work is its comprehensive vision of the healthcare environment since it involves multiple types of caregivers and structures. In this case, a possible limitation is the missing of physicians and clinical engineers among our participants, potential subjects for future studies. Moreover, it would be useful to repeat the procedure focusing on design suggestions specific to every environment. More interestingly, the same study could be performed with patients, offering their point of view and confirming our results about the caregivers’ perception of their needs. Finally, all participants were Italian caregivers, which makes the study not fully generalizable.

However, following our results, we believe that future designers could create beds that significantly impact the caregivers’ work. In our vision, the medical bed should represent a proactive and reliable instrument, able to help caregivers and make their work easier and lighter.

## Figures and Tables

**Table 1:** Table describing the characteristics of the participants participating in the study.

FG Code	Current Work Organization	Age		Gender		Healthcare Experience (years)		Past Experience in Different Healthcare Facilities		Electrical Bed Experience (years)	
		Mean	SD	F	M	Mean	SD	Yes	No	Mean	SD
FG1	Hospital	38.83	12.34	5	1	15	9.78	6	0	1.5	0.55
FG2	Hospital	41	9.93	2	2	16.8	13.62	3	1	15.25	9.81
FG3	Elderly Retirement Home	41.8	7.33	4	1	20.4	8.59	4	1	9.4	6.84
FG4	Institution for people with disabilities	33.6	70.69	4	1	8.4	5.13	1	4	8.4	5.13
FG5	Hospital	44	8.19	2	1	7	7.81	3	0	7	7.81
FG6	Domiciliary home care	35	4.82	2	4	11	5.06	5	1	9.33	5.65

**Table 2:** UX constructs emerging from the study previously explored in the literature.

Safety [97]	Refer to the users’ perception to minimize the levels of risk using the system
Comfort [98]	The extent to which the user is satisfied with physical comfort using the system
Ease of Use [99]	Refers to the degree to which the user believes s/he can use the system effortlessly
Timesaving [100]	The degree to which a person perceived itself as able to accomplish her/his objectives in a reasonable amount of time using the system
Workload [101]	The total cognitive load, or amount of mental processing power needed to use a system
Perceived Usefulness [102]	The degree to which a person believes that use of a particular system would enhance his or her job performance
Flexibility [98]	Measure of the extent to which the system is usable in all potential contexts of use
Aesthetic [103]	Refer to the capacity of a system to pleased one or more of our sensory modalities.
Reliability [104]	Refer to the users’ perception that the tool assesses the consistency

## Data Availability

The data presented in this study are available on request from the corresponding author. The data are not publicly available due to privacy issues.

## Appendix A

**Bed Element**	**Desires**	**Description**	**N°of Occurrences during Interviews**
**Side Rails**			
	Split into two sections	Pro: Easy to clean, escape routes for tubes and drains, less evident restraint, follow backrest movement. Cons: The patient can get stuck in empty spaces.	10
	Unique	Pro: Quickly lowered with a single gesture, avoid that cables and patient stuck between the said rails Cons: Possible patient joint between the side rails boards	6
	Low	Less dangerous falls	5
	Adjustable	Both electrically and manually	4
	Support points	To permit lean of the patient	3
	Curved, rounded	Less sense of restraint and suffocation	6
	Release mechanism that slides under the bed net	Avoid accidents, avoid creating gaps between the bed and other supports	8
	Easy to use and handy	They help in making the bed	3
	Single action to unlock		4
	One-hand release		3
	Quickly closable		1
	Sides lowered alarm		1
	Braked	Avoid pinching	1
	Light		2
	Resistant		5
	Soft inside / padded	Safe and comfortable	7
	Made of plastic		1
	Fireproof		1
	Smooth		2
	Solid/Transparent sides and non-straight bars	They prevent patients’ feeling of imprisonment	7
**Headboard/Footboard**			
	Not bulky		2
	Flexible for accessories		2
	Removable	Facilitate the positioning of bulky equipment, feature that gives comfort and safety	4
	Light		3
	Soft	Avoid injury to the patient	2
	Quick to release	Comfortable for hygiene and therapies	3
**Bed Surface**			
	Solid surface	Facilitating cleaning as it is a surface that tends to get dirty	2
	Waterproof		1
	Smooth		1
	Resistant		1
	Solid		1
**Electrical System**	Multi-socket for instrumentation	Solve the cable jam problem	5
	Internal multi-socket bed for the patient		1
	Head side socket		2
	Magnetic socket		1
	Integrated anti-decubitus mattress plugs		1
	Integrated electrical socket		3
	Integrated USB port		2
	Hidden electrical cables	They wear less and improve the aesthetics of the bed	2
	Socket bed disconnection alarm		1
	Battery autonomy	It allows to use the functions of the bed even when you are disconnected from the socket	1
	Auxiliary Battery	Useful in case the bed runs out of current	1
**Accessories**			
	Bed flexibility to accommodate different types of accessories	IV pole integrated into the bed and adaptable	4
IV pole			
	IV pole	Comfortable and useful	4
	Integrated into the bed and adaptable	It can be used while on the move	5
	Robust	It would solve the problem of poles breaking too easily	2
Attack for diuresis			
	Raised or adjustable connection	It would solve the problem of the pockets that touch the ground	6
Support surfaces			
	Integrated support surface to support the instrumentation		4
storage compartment			
	Integrated		4
Restraint anchors			
	Integrated	Comfortable and remove the risk of harm to the patient	4
Mattress			
	Adequate size to the bed	To cover empty spaces and prevent them from slipping	4
Triangle pole			
	Removable	It would reduce the danger to the patient when this tool is not needed	3
Headrest			
	Integrated	Help for patient hygiene but also an element of comfort	2
Bed-wall spacer			
	Integrated	It would avoid bumps	2
Blankets Lifter			
	Integrated		3
	Electrically adjustable		2
Bumper wheels			
	Space-saving		1
Armrest			
	Integrated		1
Connection for the Anti-decubitus mattress			
	Integrated		1
Bed-stretcher transfer system			
	Systems to facilitate the movement of the patient from the bed and stretcher	They should avoid the empty space between the two	5
Bed Base			
	Flexible	It would help with regards to the use of the patient lift trolley	1
**Bed Height**			
	Ability to adjust the height	Indispensable for comfort and operator facilitation	9
	Minimum height	Important for patient safety	2
	Quite wide height adjustment range		4
	Increased height adjustment speed	Pro: it helps the staff work Cons: may confuse the patient	2
	Automatic height adjustment		1
	Autonomous height adjustment by the patient	Psychologically it makes the patient feel more involved	1
**Patient Postural Management**			
	Handling support	Reduce patient discomfort	1
	An element of peace of mind for the operators	Adapt the tool to your needs	1
Inclinometer			
	Useful	0 ° indication	1
	Position	At the foot part of the bed	1
	Inclination of the entire bed	Avoid slipping (Trendelenburg)	1
	Degrees of inclination	Ability to accurately set the inclination, visible degrees (not just 30 °)	1
	Reliability	No ball, light by degrees of inclination	1
Sections			
	Increase number	Adaptable to different stature, multiple postures, patient comfort	3
	Handling	Reduces fatigue, promotes patient independence	3
	Increase movement speed	Usage time reduction	6
	Accompanied movements	Avoid patient disorientation	2
Trendelenburg			
	Useful		4
	Patient movement	Towards the headboard or footboard, solve the sliding problem, reduction of staff fatigue	4
	Automatic	Automatic patient repositioning	1
	Minimum height		1
Side tilt			
	Lateral patient repositioning	Prevent pressure sores, facilitate the insertion of aids	5
Fohler			
	Patient feeding	It favors autonomy and comfort	5
Bed exit support			
	Method for straightening patient		2
	Allow sitting position	Therapy, patient comfort, operator convenience	3
Posture Alarm			
	Posture alarm	Report the need for posturing to staff	1
Proposals for new functions			
	Standard	Preset position reset, via a button	1
	Electric leg movement		1
	Wave movement of the sections	Patient re-positioning	1
**Bed Commands**			
	Push-button panel integrated into the sides	Avoid wire stuck in the sides, avoid frequent falls (breakage of hooks)	3
	Patient control lock	Maintain posture for therapy	4
	Magnetic coupling		1
	Unified tool	Same remote control for operator and patient, same remote control for all beds	1
	Wireless		2
	Usable with feet	Avoid problem of busy hands	1
	Vocal commands	Hygiene	1
	Touch control panel	Avoid fret wear	1
	Controls on mobile app		1
Buttons			
	Easy to use		5
	Intuitive	Lighter icons, help	5
	Familiars	Referable to a domestic context, highlighting potential bed	1
	Reliable		2
	Readable		1
	Big		1
	Soft		1
	Not too flat		2
	Wear resistant		3
	Command speed	Too slow	2
	Reduce number of keys per patient		3
	Keyboard backlight	It does not have to bother	2
	Improve remote control grip		3
	Sound feedback on pressure		3
Button panel materials			
	Wear resistant	Icon color, shock resistant	3
	Resistant to disinfectants		1
	Rubber cover		2
	Cleanable		1
Button panel functions			
	Distinction between operator and patient commands		2
	Reduced number of commands per patient		2
	Possibility of customization		1
	Ability to use multiple functions at the same time		3
	Replaceable	In case of maintenance	1
**Nurse Call**			
	Microphone to speak to the guardhouse	Understanding call priorities, avoiding unnecessary interventions	2
	Video/monitor		1
	Vocal command		1
	Integrated		1
	Urgency alarm for operators		1
**Patient Parameters**			
	Parameter monitoring through integrated tools		5
	Integrated monitor	View vital signs	4
	Integrated scale (weighing)		2
	Catheter bag weight sensor		3
Patient parameter alarms			
	Bed exit		4
	Agitated patient		1
**Bed Size**			
	Extendable bed (or bed extension)	Adapt to the patient’s height	8
	Larger beds	Increase patient comfort, especially for obese patients	4
	Adaptable width (or bed spreader)		1
	Narrower bed	To facilitate movement	2
	Avoid incompatibility between bed and mattress	Avoid gaps and reduce mattress wear	4
**Bed Weight**			
	Minimized	Encourage travel	8
	Lightweight removable components		1
	Importance of the weight lifted by the bed	Easier to lift heavy patients	4
**Maneuverability**			
	Bed size proportionate to the environment (doors)		1
	Lighter bed promotes maneuverability		5
	Weight helps for displacements		1
Wheels			
	Improve maneuverability		6
	Improve wear resistance		1
	Double driving mode	Lock front wheel rotation	1
	Retractable wheels	Lower height, less hospital look	2
	Addition of a motorized wheel	Travel support	2
	Shockproof material	Comfort	1
Brakes			
	Possibility of locking all the wheels with a single brake		2
	Brake accessible for operator		1
**Materials**			
	Smooth		2
	Robust		7
	Easy to clean		10
	Plastics		4
	Light	For removable components	1
	Wear-resistant paints	For patient safety and aesthetic factor	5
	Reduce edges and cracks for hygiene		4
**Maintenance**			
	Manual controls in case of failure	To be able to use the bed in an emergency	9
	Ability to remove individual components for maintenance	Avoid replacing the entire bed in case of maintenance	3
Maintenance alarms			
	Malfunctioning sensor	Failure reporting	1
	Automatic alarm in case of failure	Automatic call for assistance	1
**Aesthetics**			
	Domestic look, less hospital	Patient comfort, familiar look	4
	Modern look		1
	Roundish		1
	Hidden mechanical parts		1
	Retractable wheels		1
	Colorful or oddly shaped beds for children		4
**Colors**			
	Warm and relaxing	Avoid strong colors	8
	Shades of color		1
	Color that integrates with the room		2
	Faux wood		6
	White color (avoid)	Cons: More prone to getting dirty	2
**Lights**			
	Night light	Patient wellbeing, support for operators	5
	Courtesy light		2
	Catheter bag illumination		2
	Bed exit light		3
	Interior lights	More domestic and welcoming environment	2
	Low intensity lights	Avoid disturbing patients	4
**Patient Relaxation**			
	Screen for video calls		1
	Integrated TV stand		1
	Integrated audio system		4
	Intercom for children		1
